# The influence of cardiac synchronisation on self-attribution to external objects in male participants

**DOI:** 10.3389/fpsyg.2024.1442942

**Published:** 2024-08-30

**Authors:** Hiroshi Shibata, Tokiko Harada, Hideki Ohira

**Affiliations:** Department of Informatics, Nagoya University, Nagoya, Japan

**Keywords:** interoception, self-attribution, sense of self, cardiac feedback, interoceptive accuracy, approach-avoidance

## Abstract

Interoception, the representation of our bodily state derived from physiological signals, is fundamental to our sense of self. Previous studies using cardiac feedback paradigms demonstrated interoceptive effects on self-perception. However, it remains unclear whether interoceptive information can extend self-attribution to non-bodily objects. This study aimed to elucidate whether cardiac signals can induce self-attribution to non-bodily objects and how interoceptive accuracy modulates this effect. A total of 44 male volunteers participated in an emotion assignment task where they viewed images of palms (bodily targets) and spheres (non-bodily targets) flashing in or out of sync with their heartbeat and assigned emotional images (positive/negative) to these targets. A heartbeat discrimination task was used to measure the interoceptive accuracy. The results showed no significant effect of synchronisation on emotion assignment for either the target type or the valence of the emotional images. However, participants with high interoceptive accuracy attributed both positive and negative images more to synchronised targets than those with low interoceptive accuracy. These findings suggest that although cardiac synchronisation may not uniformly facilitate the self-attribution of external objects, interoceptive accuracy may mediate attention to synchrony. Future studies should explore the conditions under which cardiac signals influence self-attribution.

## 1 Introduction

Interoception refers to the representation of the internal world, encompassing the mechanisms through which an organism detects, interprets, integrates, and regulates internal signals derived from physiological processes (Chen et al., 2021). Although we may not be consciously aware of it, interoception significantly influences various aspects of our lives such as emotions and cognition (Critchley and Garfinkel, 2017, 2018). Furthermore, interoception dysfunction is recognised as a factor in psychiatric disorders (Khalsa et al., 2018), thus highlighting the importance of understanding interoception to improve quality of life.

Interoception is considered a critical factor in the development of the sense of self (Seth, 2013; Tsakiris, 2017). Here, we generalise the sense of self as a process of self-other attribution. Humans distinguish between sensory information related and unrelated to themselves, attributing bodies, actions, and objects to either themselves or others. As interoception involves information derived from one’s own body, interoceptive signals can facilitate self-attribution (Palmer and Tsakiris, 2018). The theoretical model of the sense of self proposes multisensory integration between exteroception (e.g., vision and touch) and interoception (Tsakiris, 2017; Park and Blanke, 2019). Empirical evidence supports this hypothesis: individuals with high interoceptive accuracy are less likely to perceive a rubber hand with synchronised touch as their own hand (Tsakiris et al., 2011). Additionally, heartbeat-evoked potentials in the electroencephalogram (EEG) representing central cardiac processing co-vary with the bodily self (Park et al., 2016). These studies suggest that interoception plays a crucial role in attributing our body to ourselves.

Further evidence for the relationship between interoception and the sense of self has been obtained using the cardiac feedback paradigm. Cardiac feedback involves synchronising or asynchronising participants’ real-time heartbeats with stimuli, such as changing the colour and size of these stimuli. Such stimuli can affect perception. For instance, asynchronous stimuli elicit longer looking times in infants (Maister et al., 2017; Imafuku et al., 2023) and monkeys (Charbonneau et al., 2022), while older infants and adults exhibit longer looking times for synchronous stimuli (Imafuku et al., 2023; Tünte et al., 2023). The perception of changing flashes in response to a neutral stimulus differs depending on the synchrony of the flash with the heartbeat (Azevedo et al., 2016). Some studies have illustrated the interoceptive role for sense of ownership (Aspell et al., 2013; Suzuki et al., 2013; Heydrich et al., 2018, 2020). Suzuki et al. (2013) applied cardiac feedback to the rubber hand illusion and demonstrated that ownership of the rubber hand was induced when it flashed synchronously with the participants’ heartbeats. The same paradigm was used for the full-body illusion in which participants attributed a virtual body that visualised their heartbeat to themselves (Aspell et al., 2013). Another study found that individuals were more likely to identify morphed faces of themselves and others as their own when the face flashed synchronously with their heartbeat (Sel et al., 2017). Heartbeat-synchronised facial stimuli also affect reaction times for self-recognition (Ambrosini et al., 2019). These results suggest that heartbeat-synchronised signals facilitate self-attribution to both body and facial stimuli. However, it remains unclear whether the synchronisation of stimuli also elicits self-attribution in non-bodily stimuli, such as geometric objects. Thus, investigating body-specificity is essential to understanding the processes underlying the interoceptive role in self-attribution.

We can attribute the self to non-bodily objects. For example, in tool embodiment, we perceive non-bodily objects as part of ourselves through sensorimotor learning (Maravita and Iriki, 2004). In daily life, we distinguish whether an external object belongs to us based on sensory or cognitive factors. Sensory factors include the controllability of an object or multisensory contingencies, while cognitive factors involve narrative memory. However, it remains unknown whether cardiac feedback influences the self-attribution of an object. Self-attribution to the body and to objects might involve different processes. For instance, the rubber hand illusion becomes weak or absent when the fake hand is unlike a real hand (Tsakiris and Haggard, 2005; Haans et al., 2008). Conversely, some studies imply that the interoceptive effect on self-attribution may involve less body-specificity in visual-respiration and cardiac synchrony (Aspell et al., 2013; Adler et al., 2014). However, the reduced body-specificity observed in a previous visual cardiac synchrony study (Aspell et al., 2013) was not directly linked to self-attribution but rather to the localization of tactile stimuli. Therefore, it is necessary to clarify whether cardiac feedback influences the self-attribution to an object. Additionally, as responses to direct questions may include demand characteristics (Lush, 2020), it is necessary to develop a new method for implicitly assessing participants’ sense of self regarding objects.

This study investigated whether cardiac signals can induce self-attribution to bodily and non-bodily targets. To measure the implicit sense of self towards targets, we developed a new experimental task called the emotion assignment task. In this task, participants viewed target images of a palm (i.e., a bodily target) or sphere (i.e., a non-bodily target) on a screen that flashed in or out of sync with their heartbeat and assigned emotional images (i.e., positive and negative) to the targets. According to approach-avoidance theory, humans tend to approach positive things and avoid negative things (Elliot and Covington, 2001; Eerland et al., 2012; Saraiva et al., 2013). For example, Saraiva et al. (2013) demonstrated that reaction times were generally faster when approaching positive stimuli and avoiding negative stimuli in tasks involving self-relevant mannequins. Therefore, we hypothesised that participants would unconsciously assign positive and negative images to targets attributed to and not attributed to themselves, respectively. Two hypotheses were considered: first, that self-attribution would be induced, even for non-bodily targets, and second, that cardiac signals would only affect bodily targets. Additionally, we investigated whether these results were influenced by interoceptive accuracy, as measured by a modified heartbeat discrimination task (Whitehead et al., 1977). Based on a previous study (Suzuki et al., 2013), we predicted that participants with high interoceptive accuracy would promote self-attribution of synchronised stimuli.

## 2 Materials and methods

### 2.1 Participants

The participants were 44 healthy male students (*M* = 21.24 years, *SD* = 2.45) attending Nagoya University in Japan. The sample size was determined based on an *a priori* power analysis. A medium effect size (*f* = 0.2) was assumed, with an alpha level of 0.05 and a desired power of 0.80. The analysis indicated that a minimum of 40 participants would be required to detect a statistically significant effect. To account for potential dropouts, we recruited 44 participants. All participants were right-handed, except for two. Owing to gender differences in interoceptive accuracy (Prentice and Murphy, 2022) and brain responses to emotional stimuli (Wrase et al., 2003), only male participants were recruited. Data from three participants were excluded due to recording errors in the physiological or behavioural data, leaving data from 41 male participants for the analysis. This study was approved by the Department of Psychology Ethics Committee of Nagoya University (No. NUPSY-221003-G-01). All participants provided written informed consent before the experiment. Participants were compensated for their participation.

### 2.2 Stimuli

**Figure 1:**
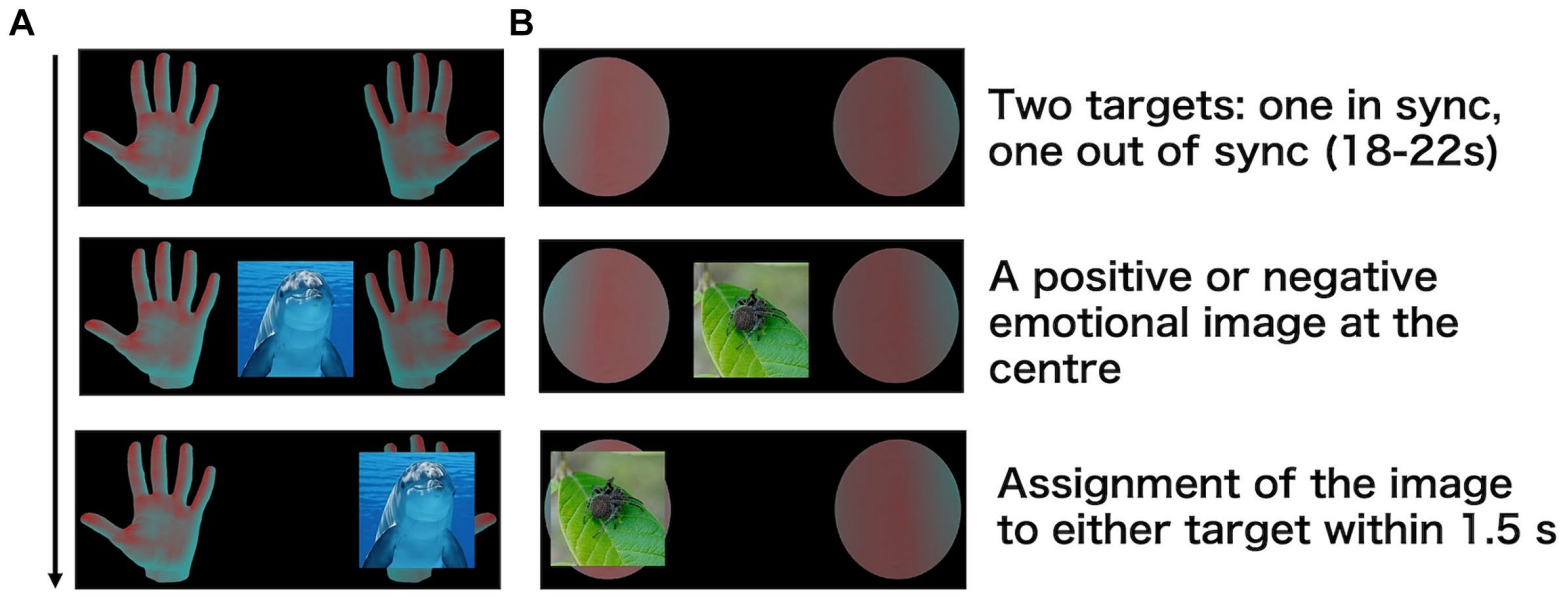
Procedures of the emotion assignment task. Participants looked at two targets [**(A)** Palm **(B)** Sphere], which were either in sync or out of sync with their heartbeat. After 18–22 s, an emotional image appeared at the centre, and participants assigned it to one of the targets.

A palm image was used as a bodily target, and a sphere image was used as a non-bodily target (Figure 1). Target images were obtained from free 3D object online sources.^1^

For the positive and negative emotional stimuli, 50 images of mammals and 50 of insects were collected from online sources.^2^^,^^3^ After aligning the illuminance of all images, 20 images of mammals and 20 of insects were selected for the current experiment based on the subjective ratings of 31 other participants in an independent evaluation experiment. Ratings were collected using an analogue scale ranging from very negative to very positive for valence ratings and from low to high arousal for arousal ratings. We selected 20 images of mammals and 20 of insects to equalise the arousal ratings. The valence ratings for images of mammals were significantly higher than those for insect images. Left–right reversed versions of the 40 images were created to counteract any left–right bias as participants positioned the images to the left or right during the task. Therefore, a total of 80 images (40 normal and 40 reversed) were used in the task.

### 2.3 Task

#### 2.3.1 Heartbeat synchronisation

During the self-attribution and heartbeat discrimination tasks, electrocardiogram (ECG) data were collected for real-time cardiac feedback. The ECG data were recorded using an MP160 system with an ECG100C amplifier (Biopac) and AcqKnowlege-NDT software. The luminance of the target stimuli (i.e., palm and sphere) on the screen gradually increased up to +40% from R-wave to R-wave +250 ms and decreased from R-wave +250 ms to R-wave +500 ms. In the synchronous condition, the participant’s real-time heartbeat was used, whereas in the asynchronous condition, the participant’s heartbeat from 60 s earlier was used. In the asynchronous condition, we did not change the heart rate to avoid differences in total luminance between the conditions.

#### 2.3.2 Emotion assignment task

Participants were presented with the two target images (i.e., two palms or two spheres) on the left and right sides of the screen (Figure 1). The screen was positioned 57 cm away from the participants, and the targets were within a 15° view angle. The luminance of one of the two images changed synchronously with the participant’s heartbeat, whereas that of the other changed asynchronously. After 18–22 s, an emotional image appeared at the centre of the screen. The duration was determined on the basis of a previous study that showed that ownership is established in approximately 20 s (Finotti et al., 2023). Participants were instructed to intuitively assign the emotional image to either the left or right target within 1.5 s. Participants used their right index and middle fingers to respond. They were not informed whether the luminance changes of the target stimuli were synchronous or asynchronous with their heartbeat. The experiment consisted of 80 trials, with the target stimuli being palms in half of the trials and spheres in the other half. This task was conducted using a block design, with palm or sphere stimuli presented consecutively in the first half, followed by the other stimulus type in the second half. The order of the stimuli was counterbalanced across participants. Participants were allowed to take a break every 20 trials.

#### 2.3.3 Heartbeat discrimination task

To determine whether participants could explicitly discriminate between targets flashing in sync and not in sync with their heartbeat and to calculate interoceptive accuracy, a heartbeat discrimination task was conducted after the emotion assignment task. Participants were presented with two flashing stimuli for 18–22 s, one of which was synchronised with their heartbeat and one was not. As in the attribution task, the synchronous stimuli were set to the real-time participant’s heartbeat, while the asynchronous stimuli were set to the participant’s heartbeat from 60 s prior. Subsequently, they were asked to judge which stimulus was synchronised with their heartbeat. The task consisted of 40 trials, with 20 involving spheres and 20 involving palms. This task was conducted using a block design, with palm or sphere stimuli presented consecutively in the first half, followed by the other stimulus type in the second half. The order of the stimuli was counterbalanced across the participants. No feedback was provided to participants regarding their performance.

### 2.4 Procedure

Prior to the experiment, participants rated 40 emotional images using a visual analogue scale to measure their subjective arousal and valence online. The valence rating ranged from 1 (very negative) to 7 (very positive), and the arousal rating ranged from 1 (very low) to 7 (very high). After completing the practice trials, participants performed an emotion assignment task. They were then informed that the stimuli were either synchronous or asynchronous with their heartbeats and completed the heartbeat discrimination task. Finally, participants were informed of the study’s aim and asked to answer some questions. The entire procedure took approximately 90 min.

### 2.5 Data analysis

We calculated the correct rate of the heartbeat discrimination task to determine the interoceptive accuracy. As the correct rates for the heartbeat discrimination task were not normally distributed, Wilcoxon signed rank tests were applied. Participants were divided into high and low interoceptive accuracy groups based on a binomial test of each participant’s correct rate. The sync-assignment rates for the emotion assignment task were normally distributed, except for one condition (negative and sphere). A three-factor analysis of variance (ANOVA) was performed with picture valence (positive vs. negative), target type (palm vs. sphere), and interoceptive accuracy group (high vs. low) as the independent variables and the rate of assigning emotional images to heartbeat-synchronised stimuli as the dependent variable. Additionally, we examined whether participants’ attribution responses (sync/async) were influenced by image ratings (arousal and valence) using a logistic regression analysis. Other factors potentially affecting the results were also explored, including differences in the number of flashes (luminance changes) between synchronous and asynchronous stimuli and participants’ tendencies to assign emotional images to the left or right side. All analyses were conducted using the R software (version 4.3.1).

## 3 Results

### 3.1 Image ratings

The mean valence ratings were 5.49 (*SD* = 0.37) and 2.80 (*SD* = 0.47) for positive and negative images, respectively. The mean arousal ratings were 2.87 (*SD* = 0.32) and 3.50 (*SD* = 0.50) for positive and negative images, respectively.

### 3.2 Heartbeat discrimination task

**Figure 2:**
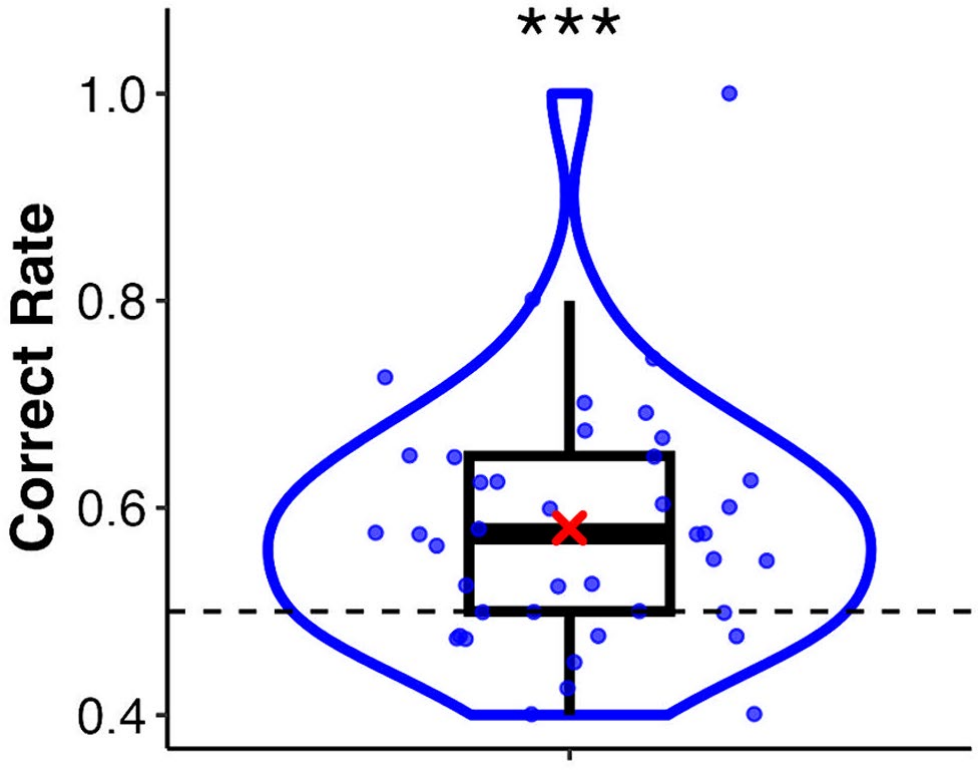
Box and violin plots for the correct rate of the heartbeat discrimination task. The asterisk indicates a significant difference from the chance level of 0.5. ***p < 0.001.

In this task, the mean correct rate was 0.58 (*SD* = 0.12). A one-sample Wilcoxon signed-rank test showed a significant difference from the chance level of 0.5 (*V* = 4.45, *p* < 0.001, *r* = 0.62) (Figure 2). The mean correct rates for the palm and sphere conditions were 0.57 (*SD* = 0.15) and 0.59 (*SD* = 0.13), respectively. The Wilcoxon signed-rank test showed no significant difference between the two conditions (*V* = 231, *p* = 0.38, *r* = 0.14). A binomial test revealed that 11 of the 41 participants discriminated the synchronised target significantly (*p*-values ranged from 0.00 to 0.04, see Supplementary Table S1). These participants were classified into the high interoceptive accuracy group, whereas the remaining participants were classified into the low interoceptive accuracy group.

### 3.3 Emotion assignment task

#### 3.3.1 ANOVA

**Figure 3:**
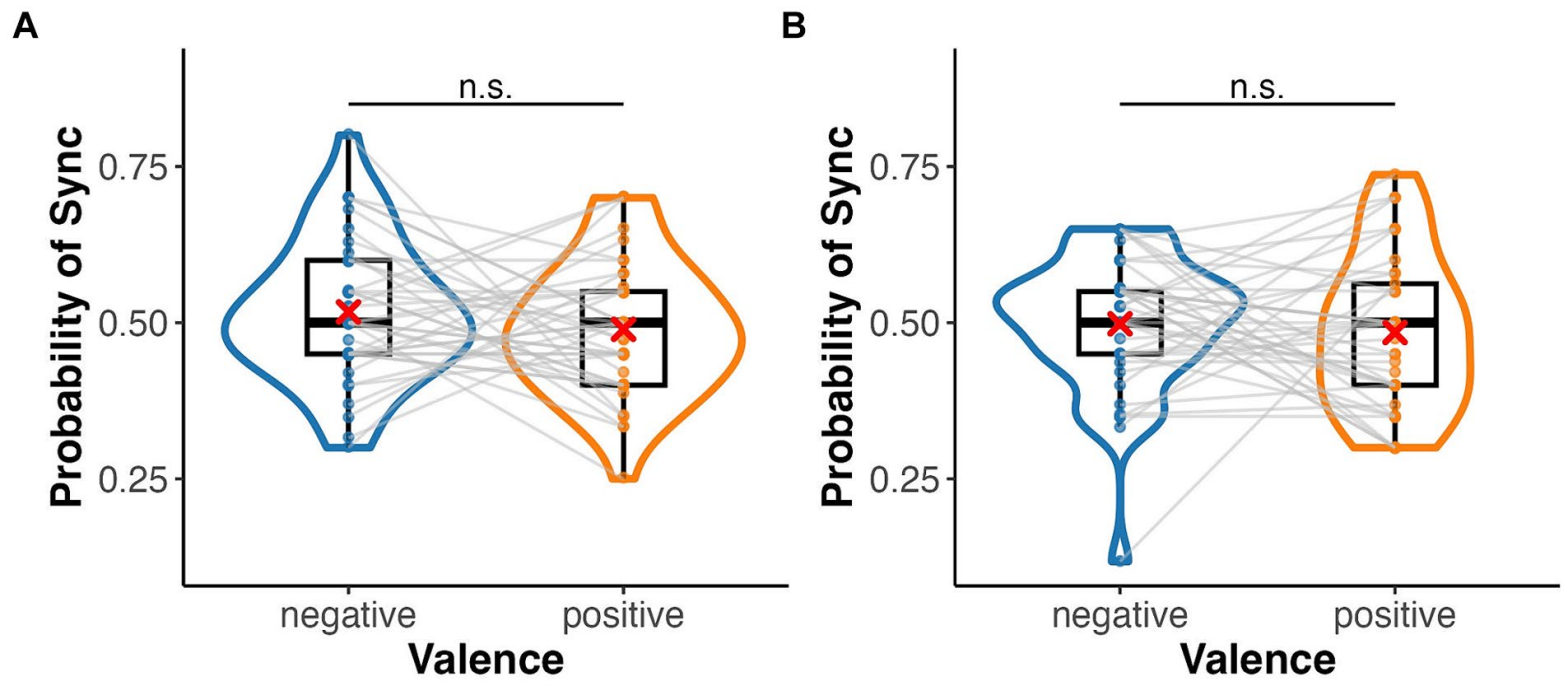
Box and violin plots for the rate of assigning an emotional (positive/negative) image to the synchronous target in the emotion assignment task. **(A)** Palm (bodily) target and **(B)** sphere (non-bodily) target.

**Figure 4:**
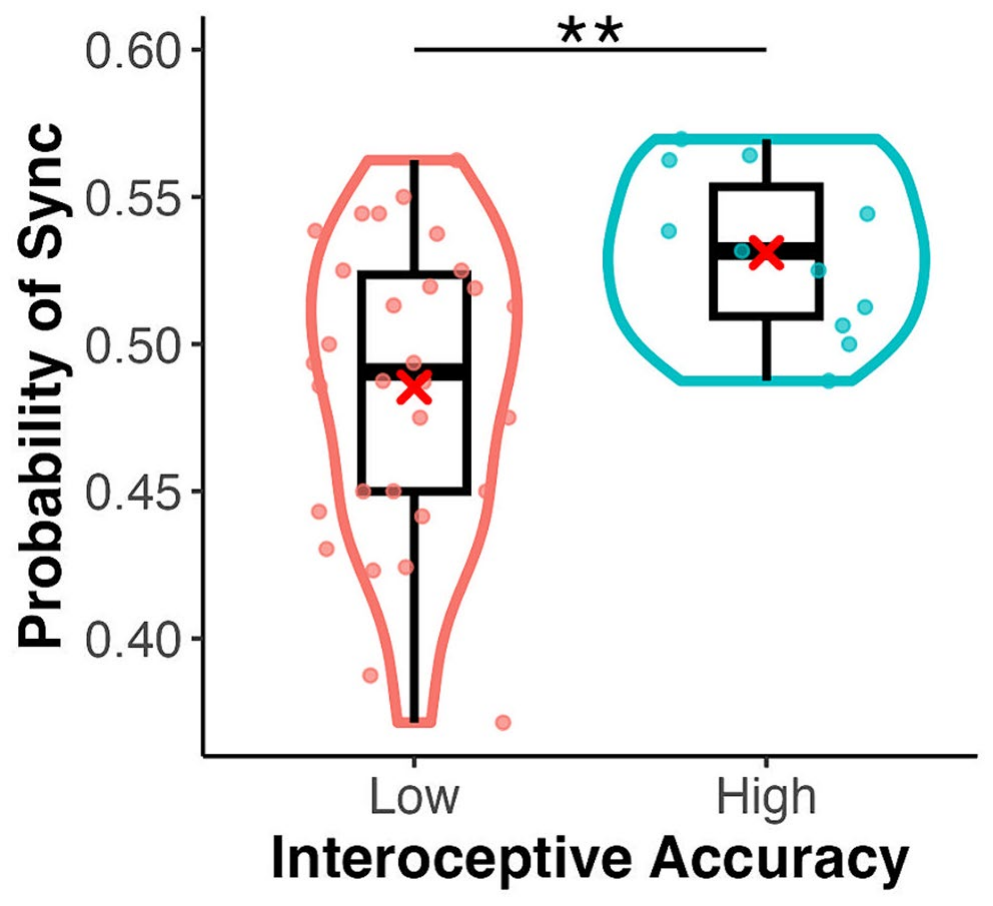
Box and violin plots for the rate of assigning an emotional image to the synchronous target by interoceptive accuracy group (high/low) in the emotion assignment task. **p < 0.01.

In the emotion assignment task, the mean rate of assigning positive and negative images to heartbeat-synchronised targets was 0.49 (*SD* = 0.11) and 0.52 (*SD* = 0.11), respectively, in the palm condition, and 0.48 (*SD* = 0.12) and 0.50 (*SD* = 0.11), respectively, in the sphere condition (Figure 3). The mean sync-assigning rates in high and low interoceptive accuracy groups were 0.53 (*SD* = 0.09) and 0.48 (*SD* = 0.12), respectively. A three-factor ANOVA showed no significant main effect of emotional images (*F*(1, 39) = 0.35, *p* = 0.556, ${\eta_{p}}^{2}$ = 0.01) or target type (*F*(1, 39) = 0.02, *p* = 0.88, ${\eta_{p}}^{2}$ = 0.00) but a significant main effect of interoceptive accuracy group (*F*(1, 39) = 8.42, *p* = 0.006, ${\eta_{p}}^{2}$ = 0.18) (Figure 4). There were no significant interactions between emotional images and target type, between emotional images and interoceptive accuracy, or between target type and interoceptive accuracy (*F*-values ranged from 0.46 to 1.20, *p*-values from 0.28 to 0.70). The three-way interaction was also not significant (*F*(1, 39) = 0.88, *p* = 0.35, ${\eta_{p}}^{2}$ = 0.02). A one-sample t-test showed a significant difference from the chance level of 0.5 in the high interoceptive accuracy group (*t*(10) = 3.65, *p* = 0.004, *d* = 1.10), whereas there was no significant difference from the chance level in the low interoceptive accuracy group (*t*(29) = −1.66, *p* = 0.11, *d* = −0.30).

#### 3.3.2 Logistic regression analysis

Based on participants’ responses to image ratings, we analysed the effect of images on the responses (synchronic/asynchronous) using logistic regression. The results suggested that neither valence nor arousal ratings significantly influenced the likelihood of participants’ responses being sync or async. Specifically, neither valence rating (*β* = 0.02, *p* = 0.40) nor arousal rating (*β* = 0.00, *p* = 0.83) were significant predictors of participants’ responses. However, interoceptive accuracy, which indicates the correct rate of each participant for the heartbeat discrimination task, was a significant predictor (*β* = −0.74, *p* = 0.018). This indicates that participants who performed well on the heartbeat discrimination task were more likely to have synchronous responses.

#### 3.3.3 Exploratory analysis

We conducted an exploratory analysis to identify other factors that may have affected the results. According to the logistic regression model, the difference in the number of flashes between sync and async targets affected the sync-assigning rate (*β* = 1.27, *p* = 0.03). Participants were more likely to assign images to targets that flashed more frequently. Additionally, participants were significantly more likely to assign positive images to the right side and negative images to the left side, although the total numbers of left and right assignments were not significantly different (Table 1). Pearson’s chi-square test indicated a significant association between emotional valence (negative/positive) and direction (left/right) ( $X^{2}$ (1) = 95.36, *p* < 0.001), although a binomial test did not show a significant difference in the total number of left and right assignments (*p* = 0.72).

**Table 1:** Number of emotional images (positive/negative) assigned to the left and right.

	Left	Right	Total
Negative	935 (796)	668 (807)	1,603
Positive	658 (797)	946 (807)	1,604
Total	1,593	1,614	3,207

Expected frequencies are shown in parentheses.

## 4 Discussion

This study examined whether cardiac signals can induce self-attribution to non-bodily and bodily targets. To measure implicit self-attribution to external stimuli, we developed a new task called the self-other-attribution task. Our main findings were as follows: (1) participants did not exhibit a significant relationship between valence and synchrony in either the bodily (palm) or non-bodily (sphere) conditions and (2) participants with high interoceptive accuracy assigned emotional images to synchronous targets significantly more often than those with low interoceptive accuracy.

Contrary to previous studies (Suzuki et al., 2013; Sel et al., 2017), we did not find any self-attribution to cardiac-synchronous stimuli, even in the bodily stimuli. There are two possible reasons for this: (1) the experimental paradigm may not be appropriate to investigate self-attribution and (2) visual-heartbeat synchrony may not promote self-attribution.

Based on approach-avoidance theory, we predicted that participants would significantly assign positive and negative images to cardiac-synchronous and asynchronous targets, respectively; however, this tendency was not observed in the current experiment. One possibility is that a more explicit experimental condition is required to evoke an approach-avoidance response. The self-target in our experiment might have been too subtle compared to that of previous studies (Eerland et al., 2012) because the participants were completely unaware of the heartbeat synchronisation. Instead, they assigned positive and negative images to the right and left sides, respectively. This may be related to the fact that most participants were right-handed, and humans typically use their dominant hand to approach and their non-dominant hand to avoid (Casasanto, 2009; Brookshire and Casasanto, 2012). For right-handed participants, the right side was easier to access and thus connected to approach, while the left side was slightly more difficult to access and thus connected to avoidance.

One experimental difference between this study and previous studies was the way in which the asynchronous conditions were created (Aspell et al., 2013; Suzuki et al., 2013; Sel et al., 2017). For the asynchronised targets, we used the same participants’ heartbeats with a 60-s delay to highlight the differences in heartbeat timing. Previous studies on real-time heartbeat feedback have often manipulated the speed of heartbeat rather than heartbeat timing (Aspell et al., 2013; Suzuki et al., 2013; Sel et al., 2017). However, in these studies, the heart rate and R-R interval feedback in the asynchronised condition differed from those in the synchronised condition, which may have impacted the results. Our exploratory analysis showed that the difference in the number of flashes between the sync and async targets affected the sync-assigning rate. This suggests that an increased number of flashes might enhance the saliency of the stimuli, leading to greater assignment.

Another possibility is that visual-heartbeat synchrony may not induce self-attribution in visual stimuli. Indeed, several studies have reported negative results regarding self-attribution and interoception (Porciello et al., 2016; Horváth et al., 2020; Moffatt et al., 2024). For example, Porciello et al. (2016) demonstrated that visuo-cardiac synchrony does not enhance self-attribution of the face, suggesting that their negative findings could be due to stimulus differences between the face and other body parts. However, the current study found that negative results also occurred for other body parts, such as the palm. This suggests that visual-heartbeat synchrony may not promote self-attribution, or at least that visual-heartbeat synchrony alone may be insufficient.

One possible explanation is that top-down cognitive processes are necessary to induce self-attribution in objects (Tsakiris and Haggard, 2005). In previous studies, the experimental settings were more explicit, making it clear to participants that the task involved self-attribution, and the difference between the two flashing speeds may have been more noticeable (Aspell et al., 2013; Suzuki et al., 2013). This suggests that top-down processes, such as participants’ expectations and awareness, might contribute significantly to the self-attribution that arises from synchrony. In contrast, it is possible that in this experiment, participants did not consciously notice the synchronisation of the target, leading to a lack of self-attribution due to insufficient top-down processing. Another possibility is that acquiring self-attribution outside one’s personal space is challenging. In this study, unlike in previous rubber hand illusion studies where the hand was typically positioned between participants’ actual hand and the centre of their bodies, the palm was presented on a screen away from the participants’ bodies (Botvinick and Cohen, 1998; Suzuki et al., 2013). This distance may have made it difficult for participants to perceive the palm as their own. Thus, careful consideration should be given to the requirements for invoking the bodily illusion.

Participants with high interoceptive accuracy were significantly more likely to assign images to synchronous targets. This difference was not only significant compared to the low interoceptive group but also above the chance level, whereas the low interoceptive group showed no significant difference from the chance level. This result aligns with those of previous studies in which older infants and adults looked at cardiac-synchronous stimuli for longer than asynchronous stimuli (Imafuku et al., 2023; Tünte et al., 2023). Synchronous stimuli may attract attention because they are familiar to adults with well-developed heartbeat attentional systems. In our study, based on previous studies (Imafuku et al., 2023; Tünte et al., 2023), it is reasonable to assume that synchronous stimuli may unconsciously attract attention in the high interoceptive accuracy group. This kind of attention or perception of signals related to our innate state could be the basis of the sense of self, although some gaps may exist in generating self-attribution, such as whether objects are within the peripersonal space. This study provides behavioural evidence of how cardiac-synchronous signals affect our minds. Future studies should incorporate both eye-tracking and behavioural measurements to further investigate this synchronous physiological effect.

This study found that participants with high interoceptive accuracy were more likely to assign emotional images to synchronised stimuli. However, previous studies have shown a negative relationship between interoceptive accuracy and self-related measures (Tsakiris et al., 2011; Honda and Nakao, 2022). These discrepancies are thought to stem from differences in the (1) experimental and (2) interoceptive tasks. First, continuous real-time biofeedback may be necessary for individuals with a high interoceptive accuracy to experience the sense of self relative to an external object. Second, while both the heartbeat counting task (Schandry, 1981) and the heartbeat discrimination task (Whitehead et al., 1977) are considered measures of interoceptive accuracy, they exhibit only a small correlation (Hickman et al., 2020). These task differences likely explain the varied directional effects of interoceptive accuracy observed in previous studies (Tsakiris et al., 2011; Suzuki et al., 2013; Honda and Nakao, 2022). By contrast, studies using continuous cardiac feedback and discrimination tasks similar to the current study have consistently shown a positive effect of interoceptive accuracy on self-attribution (Suzuki et al., 2013). This suggests that the type of interoceptive task and inclusion of real-time biofeedback are crucial factors in determining the relationship between interoceptive accuracy and self-related measures.

There are two possible limitations in the current study. First, we recruited only male participants due to known gender differences in interoceptive accuracy (Prentice and Murphy, 2022) and brain responses to emotional stimuli (Wrase et al., 2003). Future studies should investigate whether our findings are consistent among female participants. Second, our method used participants’ heartbeats from 60 s earlier for the async condition, which may have limitations. Specifically, if participants’ heartbeats had self-similarity, both sync and async targets might have felt like their own heartbeats. To avoid this issue, future studies should shuffle the 60-s delayed heartbeats before presenting them in the async condition.

In conclusion, participants with high interoceptive accuracy were more likely to assign emotional images to synchronous cardiac stimuli. This implies that synchronous stimuli primarily attract attention in participants with high interoceptive accuracy, adding behavioural evidence to studies of preferential looking at synchronous stimuli (Maister et al., 2017; Imafuku et al., 2023; Tünte et al., 2023). However, we did not find any effect of cardiac-synchronous signals on self-attribution to bodily and non-bodily targets. We discussed the possible reasons based on approach-avoidance theory and the potential for cardiac synchronisation to evoke self-attribution. This study illuminates future studies for a more elaborate examination of the role of cardiac signals in self-attribution.

## Data Availability

The original contributions presented in the study are included in the article/Supplementary material, further inquiries can be directed to the corresponding author.

## Footnotes

^1^https://www.artec3d.com/3d-models/hand

^2^https://www.pexels.com/

^3^https://www.photo-ac.com/
