# Supplementary material for: The influence of cardiac synchronisation on self-attribution to external objects in male participants
Source: Front Psychol. 2024 Aug 30;15:1442942. doi: 10.3389/fpsyg.2024.1442942 (PMC11394186; doi:10.3389/fpsyg.2024.1442942)
Supplement: Supplementary file 1 [file Table_1.DOCX]

Supplementary Material

**Supplementary Table 1:** Results of the Heartbeat Discrimination Task, including correct trials, total trials, p-values from the binomial test, and interoceptive accuracy assignments for each participant. Participants with p-values less than or equal to .05 were classified as the "High" interoceptive accuracy group, while those with p-values greater than .05 were classified as the "Low" interoceptive accuracy group.

| ID | Correct Trials | Total Trials | P-value | Interoceptive Accuracy |
| --- | --- | --- | --- | --- |
| 1 | 29 | 40 | .00 | High |
| 2 | 28 | 40 | .01 | High |
| 3 | 22 | 40 | .32 | Low |
| 4 | 23 | 40 | .21 | Low |
| 5 | 25 | 40 | .08 | Low |
| 6 | 22 | 40 | .32 | Low |
| 7 | 20 | 40 | .56 | Low |
| 8 | 17 | 40 | .87 | Low |
| 9 | 40 | 40 | .00 | High |
| 10 | 23 | 40 | .21 | Low |
| 11 | 22 | 39 | .26 | Low |
| 12 | 19 | 40 | .68 | Low |
| 13 | 19 | 40 | .68 | Low |
| 14 | 25 | 40 | .08 | Low |
| 15 | 16 | 40 | .92 | Low |
| 16 | 19 | 40 | .68 | Low |
| 17 | 27 | 40 | .02 | High |
| 18 | 20 | 40 | .56 | Low |
| 19 | 21 | 40 | .44 | Low |
| 20 | 23 | 40 | .21 | Low |
| 21 | 26 | 40 | .04 | High |
| 22 | 27 | 39 | .01 | High |
| 23 | 16 | 40 | .92 | Low |
| 24 | 19 | 40 | .68 | Low |
| 25 | 29 | 39 | .00 | High |
| 26 | 19 | 38 | .56 | Low |
| 27 | 20 | 40 | .56 | Low |
| 28 | 26 | 40 | .04 | High |
| 29 | 24 | 40 | .13 | Low |
| 30 | 26 | 40 | .04 | High |
| 31 | 23 | 40 | .21 | Low |
| 32 | 26 | 39 | .03 | High |
| 33 | 32 | 40 | .00 | High |
| 34 | 21 | 40 | .44 | Low |
| 35 | 22 | 38 | .21 | Low |
| 36 | 19 | 40 | .68 | Low |
| 37 | 21 | 40 | .44 | Low |
| 38 | 25 | 40 | .08 | Low |
| 39 | 24 | 40 | .13 | Low |
| 40 | 23 | 38 | .13 | Low |
| 41 | 18 | 40 | .79 | Low |
